# Epigenetic Changes in Neonates Born to Mothers With Gestational Diabetes Mellitus May Be Associated With Neonatal Hypoglycaemia

**DOI:** 10.3389/fendo.2021.690648

**Published:** 2021-06-29

**Authors:** Yoshifumi Kasuga, Tomoko Kawai, Kei Miyakoshi, Yoshifumi Saisho, Masumi Tamagawa, Keita Hasegawa, Satoru Ikenoue, Daigo Ochiai, Mariko Hida, Mamoru Tanaka, Kenichiro Hata

**Affiliations:** ^1^ Department of Obstetrics and Gynecology, Keio University School of Medicine, Tokyo, Japan; ^2^ Department of Maternal-Fetal Biology, National Research Institute for Child Health and Development, Tokyo, Japan; ^3^ Department of Internal Medicine, Keio University School of Medicine, Tokyo, Japan; ^4^ Department of Pediatrics, Keio University School of Medicine, Tokyo, Japan

**Keywords:** gestational diabetes mellitus, epigenetics, DNA methylation, umbilical cord blood, neonatal hypoglycaemia

## Abstract

The detection of epigenetic changes associated with neonatal hypoglycaemia may reveal the pathophysiology and predict the onset of future diseases in offspring. We hypothesized that neonatal hypoglycaemia reflects the *in utero* environment associated with maternal gestational diabetes mellitus. The aim of this study was to identify epigenetic changes associated with neonatal hypoglycaemia. The association between DNA methylation using Infinium HumanMethylation EPIC BeadChip and neonatal plasma glucose (PG) level at 1 h after birth in 128 offspring born at term to mothers with well-controlled gestational diabetes mellitus was investigated by robust linear regression analysis. Cord blood DNA methylation at 12 CpG sites was significantly associated with PG at 1 h after birth after adding infant sex, delivery method, gestational day, and blood cell compositions as covariates to the regression model. DNA methylation at two CpG sites near an alternative transcription start site of *ZNF696* was significantly associated with the PG level at 1 h following birth (false discovery rate-adjusted *P* < 0.05). Methylation levels at these sites increased as neonatal PG levels at 1 h after birth decreased. In conclusion, gestational diabetes mellitus is associated with DNA methylation changes at the alternative transcription start site of *ZNF696* in cord blood cells. This is the first report of DNA methylation changes associated with neonatal PG at 1 h after birth.

## Introduction

Gestational diabetes mellitus (GDM) is an important perinatal problem because it can promote the development of intra-uterine foetal death, shoulder dystocia, macrosomia, and/or neonatal hypoglycaemia in offspring. A recent report indicated that epigenetic modifications associated with maternal hyperglycaemia during pregnancy can predispose offspring to develop metabolic disorders, and that the in utero environment during maternal GDM is associated with changes in foetal DNA methylation at CpG sites in genes related to metabolic function (1–7). Environmental interactions with the genome can cause epigenetic modifications, which have been investigated as mechanisms related to the development of type 1 diabetes and type 2 diabetes (8, 9). In mice, GDM alters DNA methylation in the pancreatic genome of offspring, suggesting an association with the development of abnormalities in glycolipid metabolism, type 2 diabetes susceptibility, and future obesity (10). However, no methods are available for directly measuring in utero hyperglycaemic conditions, and no associations between the degree of hyperglycaemia and methylation have been shown.

Neonates born to mothers with GDM frequently present with hypoglycaemia. Because maternal glucose traverses the placenta, fetus born to mothers with GDM develop hyperinsulinemia to decrease plasma glucose (PG) levels. However, maternal glucose is shut down after birth, which often develop hypoglycaemia in neonates who were hyperinsulinemia in utero. The presence of hypoglycaemia in neonates immediately after birth is predictive of the future development of obesity or metabolic disorders (11). Furthermore, neonates that develop hypoglycaemia, which is associated with neurodevelopmental outcomes (i.e., developmental delay at an older age), should be treated as soon as possible (12–14). Infants with hypoglycaemia can be born even to women with well-controlled GDM (15). It indicates that early-pregnancy high glucose (before GDM diagnosis) may be involved in neonatal hypoglycaemia and influence adverse foetal tissue development. Furthermore, higher maternal dietary glycaemic index and glycaemic load during early pregnancy are associated with a larger foetal abdominal circumference in late-pregnancy or offspring fat mass, even at the age of 4–6 years (16, 17). Besides, fluctuating glucose and insulin concentrations during early pregnancy are associated with childhood glucose and insulin levels (18). Therefore, neonatal hypoglycaemia may be associated with epigenetic changes in cord blood cells.

In this study, we tested the hypothesis that neonatal hypoglycaemia can reflect foetal hyperinsulinemia due to maternal early-pregnancy hyperglycaemia and that offspring cord blood epigenetics may be associated with neonatal hypoglycaemia. To this end, we examined the associations between the DNA methylation status in umbilical cord blood samples and neonatal PG at 1 h after birth.

## Materials and Methods

### Ethics Approval and Consent to Participate

The study was approved by the Keio University School of Medicine Ethics Committee (20100154, 20110321, 20150103, and 20150168) and the Institutional Review Board of the National Research Institute for Child Health and Development (406) and was conducted in accordance with the ethical standards outlined in the 1964 Declaration of Helsinki and later amendments. All subjects provided written informed consent.

### Study Population

We collected samples of cord blood from 132 offspring born at term to mothers with GDM and under neonatal care at Keio University Hospital between 2012 and 2016. Neonatal PG was routinely measured at 1 h after birth by the nursing staff, and neonatal hypoglycaemia was diagnosed according to glucose concentrations <2.6 mmol/L (47 mg/dL). GDM was diagnosed using the oral glucose tolerance test with 75 g of glucose (75 g-OGTT) according to criteria established by the International Association of Diabetes and Pregnancy Study Group (19). This required achieving one or more of the following threshold values: fasting PG, 5.1 mmol/L (92 mg/dL); 1 h PG levels during 75 g-OGTT (1 h-PG), 10.0 mmol/L (180 mg/dL); and 2 h PG levels during 75 g-OGTT (2 h-PG), 8.5 mmol/L (153 mg/dL). Each subject was evaluated based on the OGTT results. As described in our previous report, all mothers with GDM were on defined diets and self-monitored their blood glucose measurements at our hospital. Physicians analysed the blood glucose levels of all mothers every 2–3 weeks. Insulin was administered when dietary management did not result in the expected glucose levels (i.e., fasting PG level <5.6 mmol/L [100 mg/dL] or 2 h PG <6.7 mmol/L [120 mg/dL]) (20). The exclusion criteria included multiple pregnancy, preterm birth (gestational age at delivery <37 weeks), hypertensive disorder during pregnancy, neonatal asphyxia (umbilical artery pH <7.100 or Apgar score at 5 min <7), foetal growth restriction as defined by the International Society of Ultrasound in Obstetrics and Gynaecology, and congenital foetal anomaly. We also excluded women with DM (i.e., type 1 or 2 diabetes) before pregnancy and overt diabetes during pregnancy. Based on standard Japanese sex- and parity-specific birth-weight percentile curves, a birth weight ≥90^th^ or <10^th^ percentile was defined as large or small for gestational age, respectively (21).

### DNA Methylation in Umbilical Cord Blood

Umbilical cord blood was collected from each neonate immediately after birth. Genomic DNA was extracted using the QIAsymphony DNA Midi kit (Qiagen, Hilden, Germany), followed by bisulphite treatment using the Zymo EZ-96 DNA methylation kit (Zymo Research, Irvine, CA, USA). The genome-wide DNA methylation status for >850K CpG sites was analysed using the Infinium MethylationEPIC BeadChip array (Illumina, San Diego, CA, USA). Methylation data were acquired using the iScan system (Illumina) as idat files and processed by the minfi and ChAMP packages (https://bioconductor.org/biocLite.R) in R (v.3.4.0; www.R-project.org). The background was corrected using the NOOB method in the minfi package (22). Corrected data were normalized by BMIQ in the ChAMP package (v.2.8.9) (23). The manifest file was annotated using “IlluminaHumanMethylationEPICanno.ilm10b2.hg19.” We removed 11,800 probes with detection P-values >0.01 in at least one sample, 3,125 probes with a bead count <3 in at least 5% of samples, and 2,894 non-CpG targeting probes. Additionally, we filtered 17,124 probes located on either the X or Y chromosome, 49 multi-hit probes (24), and 77,589 single-nucleotide polymorphism (SNP)-related probes (25) using ChAMP. This yielded 754,255 autosomal probes from 132 samples. We used the Beta-value (β), which represents the ratio of the methylated probe intensity and overall intensity (sum of methylated and unmethylated probe intensities). The cell composition of each cord blood sample (i.e., ‘Bcell’, ‘CD4T’, ‘CD8T’, ‘Gran’, ‘Mono’, ‘NK’, and ‘nRBC’) was analysed using “FlowSorted.CordBlood.450k” in the minfi package (26). We confirmed that the same sample had not been measured twice as clustering samples by using methylation levels of 1,297 probes with a minor allele frequency of the target CpG site >0.4 (**Supplementary Figure 1A**).

### Statistical Analysis

Perinatal information was retrospectively obtained from medical records at our hospital. The significance of associations between PG at 1 h after birth and each blood cell composition or each medical record estimated using the minfi package was examined by Pearson’s association test. Significant differences in PG at 1 h after birth according to the neonatal sex or effects of caesarean section (CS) were examined by Welch’s two-sample *t* test. Eventually, we included whole-cell types, except granulocytes, in addition to gestational age, CS, and neonatal sex as covariates. Additionally, we determined whether associations between DNA methylation and PG at 1 h after birth were influenced by genetic variants. We also evaluated significant 12 CpG sites according to a reference meQTL dataset described by Hannon et al. (27).

We assessed raw and normalized β values from the 132 samples using principal component analysis to exclude the outliers (**Supplementary Figures 1B, C**), resulting in exclusion of four samples. There were no differences in maternal characteristics between included and excluded cases (**Supplementary Table 1**). First, we analysed associations between neonatal PG at 1 h after birth and blood cell composition or medical characteristics by linear regression analysis. Associations between neonatal PG at 1 h after birth and cord blood DNA methylation in the 128 samples were assessed by robust linear regression (rlm) using the R MASS package [rlm(model, method=“M”, psi = psi.hampel, init = “lts”)] with White’s estimator [coeftest(rlm, vcov=vcovHC(rlm, type=“HC”))] using the R ‘sandwich’ package. The variance inflation factor of PG at 1 h after birth or each estimated cell composition was as follows: PG at 1 h after birth (1.15), ‘Bcell’ (5.56), ‘CD4T’ (33.23), ‘CD8T’ (6.19), ‘Gran’ (55.35), ‘Mono’ (5.29), ‘NK’ (2.47), and ‘nRBC’ (4.70). Because the variance inflation factor of ‘Gran’ was the largest in this study, we removed the ‘Gran’ fractions from the covariates. After removing ‘Gran’, the covariates were PG at 1 h after birth (1.14), ‘Bcell’ (1.16), ‘CD4T’ (1.47), ‘CD8T’ (1.14), ‘Mono’ (1.25), ‘NK’ (1.23), and ‘nRBC’ (1.20). Other cell types were added to the rlm model as separate covariates. Additionally, we evaluated the rlm model by adding either cell type individually. The genomic inflation factor (λ) and quantile–quantile plots were used to compare the genome-wide distribution of P-values with the expected null distribution. The P-value was adjusted by λ and used to normalize the expected proportion of false-positives in the dataset (i.e., false discovery rate) and correct for multiple testing rounds using the Benjamini–Hochberg method. For maternal and neonatal characteristics, continuous data were compared between groups using the Mann–Whitney *U*, test and categorical variables were analysed using Fisher’s exact test using JMP software (v15.0; SAS Institute, Cary, NC, USA). A *P* < 0.05 was considered to indicate significant results.

## Results

We collected PG at 1 h after birth samples from 128 term neonates born to mothers with GDM (**Table 1**). The median gestational age at delivery and birth weight were 39 weeks (range: 37−41 weeks) and 3,022 g (range: 2,352−3,834 g), respectively. Among the 128 neonates, 63 were female (49%) and 45 exhibited hypoglycaemia (36%). The median PG at 1 h after birth was 2.8 mmol/L (range: 1.2−7.6 mmol/L). Among 45 neonates with hypoglycaemia, 12 were born to mothers with GDM who received insulin therapy during pregnancy, and 24 were born to mothers with GDM whose GDM was diagnosed before 24 gestational weeks. Maternal insulin therapy and GDM diagnosed before 24 gestational weeks were not associated with neonatal hypoglycaemia (*P* = 1.00 and 0.82, respectively).

**Table 1:** Characteristics of 128 neonates born to Japanese mothers with gestational diabetes and monitored 1 h after birth.

	Inclusion Group	
	(n = 128)	
Maternal age at delivery (years)	37	(26−47)
Maternal pregravid BMI (kg/m^2^)	20.4	(16.9–32.9)
Maternal insulin use during pregnancy	33	(26%)
Maternal gestational weight gain (kg)	8.2	(-5.2–18)
GDM diagnosis before 24 gestational weeks	71	(55%)
Gestational age at delivery (weeks)	39	(37–41)
Caesarean section	45	(35%)
Female neonates	63	(49%)
Birth weight (g)	3,022	(2,352−3,834)
PG at 1 h after birth (mmol/L)	2.8	(1.2−7.6)
Hypoglycaemia (PG <2.6 mmol/L)	45	(35%)
Umbilical artery pH	7.31	(7.13–7.44)
Apgar score		
1 min	8	(7–10)
5 min	9	(7–10)
Placenta weight (g)	560	(310–910)

BMI, body mass index; GDM, gestational diabetes mellitus; PG, plasma glucose level. Data are presented as median (range) or n (%).

The association between PG at 1 h after birth and blood cell composition or medical characteristics is shown in **Table 2**. Although there was less collinearity between cell types and 1-h PG (**Supplementary Figure 2**), ‘CD4T’, ‘Gran’, and ‘NK’ were significantly correlated with PG at 1 h after birth. For medical characteristics, gestational age at birth was significantly associated with PG at 1 h after birth; however, the antepartum OGTT or metabolic features were not associated with PG at 1 h after birth. Although there was no difference in PG at 1 h after birth between female and male new-borns (2.8 mmol/L *vs.* 2.8 mmol/L, *P* = 0.63), the PG at 1 h after birth in those born *via* CS was lower than that in new-borns born *via* vaginal delivery (2.7 *vs.* 2.8 mmol/L, *P* = 0.024).

**Table 2:** Correlation between plasma glucose level at 1 h after birth and blood cell composition or medical records.

	Trend	P-value
Cell composition of blood sample		
B cell	-0.128255	0.15
T cell, CD4	-0.279375	0.0014
T cell, CD8	0.1179542	0.18
Granulocyte	0.187439	0.034
Monocyte	0.0227967	0.80
NK cell	0.2066989	0.019
Red blood cell	0.0408877	0.65
Maternal age at delivery	-0.016684	0.85
Maternal antepartum OGTT		
Fasting PG	-0.016684	0.85
1-h PG	0.119382	0.18
2-h PG	-0.090744	0.31
Fasting IRI	-0.111603	0.21
1-h IRI	0.0194941	0.83
2-h IRI	-0.057849	0.52
HOMA-IR	-0.047058	0.60
IS_OGTT_	-0.007299	0.93
Insulinogenic index	0.0995087	0.27
ISSI-2	-0.004584	0.96
Gestational age at birth	0.2761861	0.0016
Birthweight	-0.030745	0.73
Neonatal PG	1	0

OGTT, oral glucose tolerance test; PG, plasma glucose level; IRI, immunoreactive insulin; HOMA-IR, homeostasis model assessment for insulin resistance; IS_OGTT_: Insulin sensitivity index from OGTT; ISSI-2, Insulin Secretion-Sensitivity Index-2.

**Figure 1:**
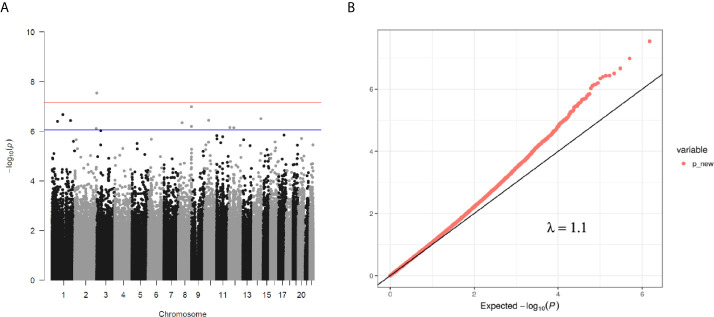
**(A)** Manhattan plot of EWAS between PG at 1 h after birth and DNA methylation levels of 754,255 CpG sites. **(B)** QQ plot of EWAS p-values.

**Figure 2:**
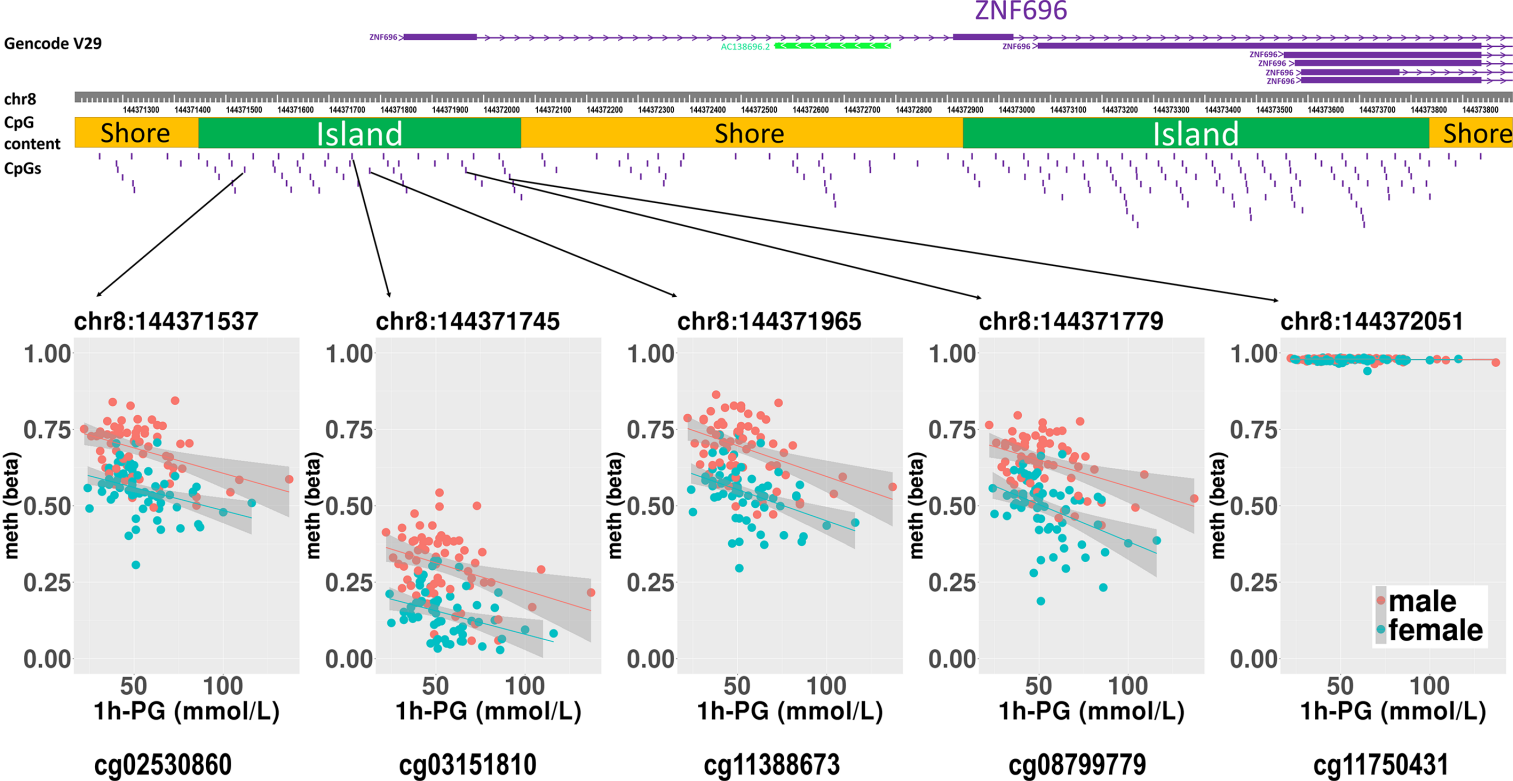
Rlm analysis of DNA methylation and 1-h PG after birth in each of five CpG sites located in the same CpG island in the *ZNF696* promoter. DNA methylation of the 5′ region of four CpG sites was associated with PG at 1 h after birth after birth. Only cg11388673 and cg08799779 were significant after considering covariates and confounders.

We then performed rlm analysis of DNA methylation of cord blood cells and continuous neonatal 1-h PG of 128 samples by adding four covariates: sex, CS, gestational age, and blood cell components. This model resulted in a λ of 1.22 (**Supplementary Figure 3**). Therefore, we corrected the P-value by adjusting λ to 1.1, which we considered as an acceptable value (28). This revealed that the methylation level at 12 CpG sites was individually associated with PG at 1 h after birth (false discovery rate-adjusted *P* < 0.05) (**Table 3** and **Figure 1**). Differences in the new-born DNA methylation beta value per 1 mmol/L increase in PG at 1 h after birth at these 12 CpG sites were -0.033–0.011. Of these 12 CpG sites, two (cg11388673 and cg08799779) were on the same CpG island (chr8:144371446-144372076) in *ZNF696* and associated with GDM according to the EWAS Atlas database. We confirmed that cell heterogeneity was not a major confounder in the associations of these two CpG sites, by adding and comparing each cell type individually as a covariate and based on the results without cell type covariates (**Supplementary Figure 4**). However, neonatal gender was a major confounder of these two CpG sites. Regression analysis between only the DNA methylation status and neonatal PG at 1 h after birth did not reveal these two CpG sites as significant (**Supplementary Table 2** and **Supplementary Figure 5**). Interestingly, previous reports have shown that these two CpG sites are associated with sex (**Table 4**) (29, 30); therefore, we examined the associations between each DNA methylation level in five array probes within the same CpG island and neonatal PG according to neonatal sex (**Figure 2**). Of the five probes, four were associated with neonatal PG at 1 h after birth in both male and female infants (*P* < 0.05) (**Table 5**). Among the four probes, sites cg11388673 and cg08799779 were significantly associated with neonatal PG at 1 h after birth according to rlm after considering the covariates and confounders (**Table 3**).

**Table 3:** Methylation sites where neonatal plasma glucose level was associated with offspring new-born blood methylation.

Target ID	CHR	Position	Coefficient^a^	SE	Raw p-value	FDR-corrected P value
cg08694578	2	241835147	0.007079794	0.001208394	4.66E-09	0.02163268
cg11388673	8	144371779	-0.033161507	0.005901354	1.92E-08	0.038674577
cg22424746	1	117753313	0.004865795	0.000888525	4.34E-08	0.042628046
cg14419205	14	103534883	0.009046023	0.001674679	6.60E-08	0.042628046
cg14126408	10	44705342	0.009221915	0.00171699	7.83E-08	0.042628046
cg26151761	1	203025885	0.004951642	0.000922286	7.92E-08	0.042628046
cg09818265	1	57917333	0.009563449	0.001787176	8.74E-08	0.042628046
cg12217831	8	40958960	0.008928346	0.001675977	9.97E-08	0.042628046
cg08799779	8	144371965	-0.032174481	0.006116658	1.44E-07	0.048566746
cg09399476	12	9838211	0.006667227	0.001272616	1.61E-07	0.048566746
cg27052152	12	55027176	0.010687028	0.002041143	1.64E-07	0.048566746
cg18158709	2	235598570	0.001918437	0.000367827	1.83E-07	0.049131149

CHR, chromosome; FDR, false discover rate; SE, standard error.

a Differences in DNA methylation beta value per 1 mmol/L increase in PG at 1 h after birth.

**Table 4:** Outputs from mQTL database and EWAS Atlas for identified 12 CpGs.

chr	CpG site	position (hg19)	position (hg38)	Annotation	Gene Name	Location	meQTL SNP	meQTL	meQTL	EWAS Atlas (We checked on August 24,2020)
							(hg38)	Beta value	P-value	
1	cg09818265	57917333	57451661	intron 3 of 16	DAB1	OpenSea				
				(NM_021080)						
1	cg22424746	117753313	117210691	intron 1 of 4	VTCN1	OpenSea				Multiple sclerosis
				(NM_001253850)						
1	cg26151761	203025885	203056757	intron 18 of 29	PPFIA4	OpenSea				IL-13 treatment
				(NM_001304331)						
2	cg18158709	235598570	234689926	intron 1 of 2	LINC01173	OpenSea				
				(NR_132376)						
2	cg08694578	241835147	240895730	exon 1 of 5	C2orf54	OpenSea				
				(NM_001085437)						
8	cg12217831	40958960	41101441	Intergenic	–	OpenSea				
8	cg11388673	144371779	143289609	Promoter-TSS	ZNF696	Island	chr8.143289197	-0.053828385	8.96E-20	Gender, GDM, Primary Sjogren’s Syndrome, Klinefelter syndrome
				(ENST 00000523891)						
8	cg08799779	144371965	143289795	Promoter-TSS	ZNF696	Island	chr8.143289818	-0.053782588	4.02E-22	Gender
				(ENST 00000523891)						
10	cg14126408	44705342	44209894	Intergenic	–	S_Shelf				Crohn’s disease, Pretem birth
12	cg09399476	9838211	9685615	intron 2 of 5	CLEC2D	OpenSea	chr12.9206399	0.01062681	2.18307E-10	
				(NM_001004419)						
							chr12.9361655	0.01149018	4.47186E-13	
							chr12.9663833	0.01600185	3.98094E-23	
							chr12.9673426	-0.04524827	2.72462E-92	
							chr12.9764510	-0.01051047	4.44425E-09	
12	cg27052152	55027176	54633392	intron 1 of 4	LACRT	OpenSea	chr12.54615029	-0.028313087	1.34E-57	Ancestry
				(NM_033277)						
14	cg14419205	103534883	103068546	Intergenic	0	OpenSea				

chr, chromosome; SNP, single nucleotide polymorphism.

**Table 5:** The association with DNA methylation at 5 CpG sites within alternative TSS of *ZNF696* and neonatal PG at 1h after birth by neonatal sex.

CGI [chr8:144371446-144372076]	Male			Female		
	Coefficient	SE	Pr(>|z|)	Coefficient	SE	Pr(>|z|)
cg02530860 (8:144371537)	-0.025	0.007	2.7.E-04	-0.027	0.007	2.7.E-04
cg03151810* (8:144371745)	-0.029	0.009	1.3.E-03	-0.029	0.008	1.6.E-04
cg11388673* (8:144371779)	-0.028	0.007	1.3.E-05	-0.043	0.010	3.3.E-05
cg08799779 (8:144371965)	-0.031	0.008	7.2.E-05	-0.036	0.010	2.1.E-04
cg11750431 (8:144372051)	-3.4.E-04	7.4.E-04	0.64	7.0.E-04	4.3.E-04	0.10

chr, chromosome; SE, standard error.

Rlm was performed adding 3 covariates; cesarean section delivery, gestational age, and cell components (-Gran).

*indicates the probes that reported to be hyper-methylated in infants cord blood born to GDM (31).

The sites cg11388673 and cg08799779 in *ZNF696* were −67 and +119 proximal to the alternative transcription start site (TSS), respectively. The transcript starting from this location (i.e., ENST00000523891) encodes a protein isoform of *ZNF696* (UniProt: E5RJV3_112 aa) with 96 amino acids of the C-terminus perfectly matching those of the N-terminus of another major isoform (UniProt: Q9H7X3_374 aa). DNA methylation in the proximity of the TSS of the major isoform (ENST00000330143) was not associated with PG at 1 h after birth (**Supplementary Figure 6**). We identified 46 transcription factors that bind the TSS of the major isoform, whereas only ELF1 and RUNX3 were confirmed to bind the alternative TSS in the human lymphoblastoid cell line GM12878 according to the ENCODE 3 TFBS Track (32), which is available in UCSC Genome Browser on Humans February 2009 (GRCh37/hg19) Assembly (**Supplementary Figure 7**). ELF1 and RUNX3 binding was not observed in any of the other 10 CpG sites.

Methylation levels at the 12 CpG sites continuously changed in association with neonatal 1 h PG level after birth. However, these epigenetic changes may be related to genetic variations concentrated in neonates in whom methylation levels at any of the 12 CpG sites were either in the higher or lower range. Evaluation of this possibility using the DNA-methylated quantitative trait locus (meQTL) database (25) confirmed the presence of eight meQTL SNPs related to methylation levels at four CpG sites out of the 12 CpG sites (**Table 4**). None of eight SNPs have been reported to be associated with any traits in the GWAS Catalog. The minor allelic frequency (MAF) of the eight SNPs was 0.290–0.490 in the Genome Aggregation Database (gnomAD; https://gnomad.broadinstitute.org). Next, to determine whether unexamined genetic variants associated with neonatal hypoglycaemia can alter methylation of the 12 CpG sites identified in our study, we referred to the GWAS Catalog. Among the six reported GDM-associated variants, none of the variants could be *cis*-meQTL within 1 Mb of the 12 CpG sites (30, 32). There were 549 significant genome-wide associations (*P* < 5 × 10^-8^) between 384 common SNPs within 1 Mb of 12 CpG sites and any trait. Of the 549 associations, SNPs associated with body mass index, fasting blood glucose, and T2DM were identified within 1 Mb of four (cg09818265, cg26151761, cg27052152, and cg14419205), one (cg12217831), and two (cg22424746 and cg14419205) of the 12 CpG sites, respectively. We cannot rule out the possibility that these meQTL- and GWAS-identified genetic variants are involved in methylation changes observed in the 12 CpG sites in a neonatal period-specific manner without evaluating the genetic variants in our subjects.

## Discussion

In this study, we measured genome-wide DNA methylation levels in umbilical cord blood and PG levels in neonates born to women with GDM. The results revealed an association between methylation at CpG sites proximal to an alternative TSS of *ZNF696* and PG at 1 h after birth.

We hypothesized that postnatal blood glucose levels reflect the degree of foetus-individual response to *in utero* hyperglycaemia, and that neonatal epigenetics and hypoglycaemia are associated. In this study, about half of the mothers were diagnosed with GDM after 24 gestational weeks. However, since we thought they might have had potential hyperglycaemia from the early stages of pregnancy according to previous reports (16–18), the DNA methylation status in cord blood cells was associated with neonatal PG at 1 h after birth. This is the first study investigating the possible association between the offspring epigenome with hypoglycaemia in neonates born to mothers with GDM. We identified two CpG sites associated with neonatal PG on the same CpG island (chr8:144371446–144372076) and in the vicinity of the alternative TSS of *ZNF696.* Moreover, another two CpG sites within the same CpG island were associated with DNA methylation and PG at 1 h after birth (**Figure 2**). A previous report indicated that a CpG site in this region is associated with GDM according to genome-wide DNA methylation analysis of offspring born to mothers with GDM (31). Although the function of *ZNF696* remains unknown, a previous study reported that this gene is involved in acquired paclitaxel resistance in nasopharyngeal carcinoma cells (33). In the present study, DNA methylation at two CpG sites within *ZNF696* showed associations with neonatal 1-h PG. Although the method used to detect DNA methylation had a limited ability to detect all CpGs in the genome, the findings using array probes suggested strong epigenetic regulation. Yamamoto et al. reported that in-target intrapartum glucose control in mothers with GDM was not associated with neonatal hypoglycaemia after adjusting for neonatal factors and considering gestational age, preterm delivery, and infant sex as confounders of neonatal factors (34). This suggests that neonatal hypoglycaemia does not occur because of temporal maternal glycaemic dysregulation during intrapartum. Therefore, the association between changes in DNA methylation identified in the present study and neonatal hypoglycaemia suggests that both epigenetic regulation and hypoglycaemia are ascribed to effects related to maternal hyperglycaemia. Furthermore, epigenetic changes to the *ZNF696* promoter may regulate blood cell function along with neonatal hypoglycaemia.

We confirmed eight meQTL SNPs, which have been reported for four CpG sites among the 12 CpG sites (**Table 4**). Whether our results were affected by these reported genetic variants and if these reported genetic variants alone were associated with the changes in the methylation at four CpG sites remain unclear. Undefined SNPs may affect neonatal methylation at the identified 12 CpG sites in *cis* or *trans* manner to synergistically interact with maternal hyperglycaemia. Further studies are needed to clarify these possibilities. The TF ChIP-seq database revealed that ELF1 and RUNX3 bind the alternative TSS of *ZNF696* based on information obtained using the human lymphoblastoid cell line GM12878 (32). These two transcription factors do not reportedly bind to other regions that are associated with the remained 10 CpG sites. This may be related to the results that showed that among the 12 significant CpG sites, methylation changes at the two CpG sites in the *ZNF696* alternative TSS alone are negatively associated with postnatal 1-h PG. Targeting of alternative TSSs is related to developmental stage (35, 36) or cell differentiation (37). This suggests that the synergistic effects of the *in utero* environment and foetal glucose tolerance are driven by DNA methylation at the alternative TSS of *ZNF696*. Furthermore, Yamamoto et al. reported that male sex was significantly associated with neonatal hypoglycaemia in children born to mothers with type 1 diabetes and GDM (34). In the present study, neonatal gender was unrelated to PG at 1 h after birth; however, we only observed an association between DNA methylation at *ZNF696* and PG at 1 h after birth when neonatal sex was added as a covariate. Moreover, hypermethylation was observed in CpG sites in the alternative TSS of *ZNF696* in males and in neonatal hypoglycaemia (**Figure 2**), suggesting differential isoform transcription at this location between genders. Similar epigenetic regulation may occur in various tissues and affect the control of blood glucose depending on the neonatal sex.

Hyperinsulinemia, insulin resistance, and metabolic syndrome reportedly alter the blood cell composition (38). We identified a significant correlation between neonatal PG and the percentage of ‘CD4T’, ‘Gran’, and ‘NK’ components in cord blood cells, respectively. This indicates that *in utero* hyperinsulinemia affects cord blood cell differentiation.

This study has several limitations. First, PG at 1 h after birth was determined only in neonates born to mothers with GDM at our hospital and not in those delivered from mothers without GDM. In addition, we did not determine the neonatal plasma insulin level. Furthermore, as neonatal hyperinsulinemia is the major reason for neonatal hypoglycaemia in offspring born to mothers with GDM, the neonatal plasma insulin level may better reflect the *in utero* environment compared to PG. However, we did not determine plasma insulin levels in neonates. To overcome this limitation, we examined the methylation beta value of cord blood from 60 new-borns born to mothers with normal glucose tolerance (NGT) but whose neonatal PG at 1 h after birth was not evaluated. Neonatal hypoglycaemia may occur not only in new-borns born to mothers with GDM but also in those who are preterm or are small or large for their gestational age (38). Our 60 NGT subjects were all born after 37 gestational weeks. Four new-borns of our 60 subjects had -1.5 SD birth weight and four had +1.5 SD birth weight compared to subjects born at the same gestational age. We predicted that neonatal hypoglycaemia had not occurred in our 60 subjects with NGT based on the following results. The methylation levels in the 60 subjects with NGT should be higher than those in 128 subjects with GDM at the 10 CpG sites whose regression coefficients for neonatal PG levels showed positive values in our analysis, and vice versa for the remaining 2 CpG sites. As shown in **Supplementary Figure 8**, the median methylation levels of NGT were higher than those of GDM at cg08694578 and cg27052152 in both sexes. Student’s *t*-test revealed significant methylation differences between NGT and GDM at cg27052152 only in males (p = 0.035). The median methylation levels at cg11388673 and cg08799779, which are in the alternative TSS of *ZNF696*, were decreased in NGT compared to in GDM as predicted, although they were not significantly different. The differences were more obvious in males. These results regarding to at least four of the 12 identified CpG sites support our speculation that methylation changes at the identified CpG sites could be markers for *in utero* hyperglycaemia, even after including NGT subjects. Further experiments are warranted to confirm these results by recruiting subjects with NGT, whose neonatal PG is evaluated at 1 h after birth. Second, the sample size was small. When calculating multiple regression power with a medium effect size and at the false discovery rate-corrected significance level, the power of our sample size was 0.024. Without adjusting the 5% significance level using multiple test correction, the power was 0.86. However, we performed association analysis between DNA methylation and neonatal PG by adding four covariates, including sex, CS, gestational age, and blood cell components. Furthermore, aside from GDM and preterm birth, maternal obesity, poor or excessive nutrition, and adiposity are associated with neonatal hypoglycaemia (39). Given that a previous study reported that umbilical cord blood was associated with PG after birth in neonates (40), analysis of the relationship between neonatal PG and DNA methylation may be more effective than examining the relationship with GDM to identify epigenetic mechanisms related to the development of neonatal hypoglycaemia. Nevertheless, further investigation using a larger cohort is necessary to identify other epigenetic mechanisms related to neonatal hypoglycaemia. We attempted to verify our results using the Asian cohort study GUSTO (41). However, in this previous study, data on cord blood DNA methylation was obtained from only 65 of 211 mothers with GDM, limiting our ability to interpret the findings in this small sample. Third, since hyperinsulinemia in mothers with GDM might be often attributed to excessive insulin therapy, we cannot deny the possibility that insulin therapy during pregnancy influenced DNA methylation in cord blood samples. However, maternal insulin therapy was not significantly associated with neonatal hypoglycaemia in our neonates. In addition, since we have introduced self-monitored blood glucose measurements for all GDM cases, we believe that extra insulin therapy did not occur in our subjects. Fourth, overt diabetes might be more strongly influenced by DNA methylation compared to GDM because maternal hyperglycaemia in overt diabetes mothers might be more prominent than that in mothers with GDM. However, in this study, only one mother developed overt DM, and we could not analyse the association between neonatal hypoglycaemia and DNA methylation in cord blood samples among neonates born to overt diabetes mothers. We have to consider further research using larger samples.

In summary, we identified DNA methylation at two CpG sites near an alternative TSS of *ZNF696* and found that they were significantly associated with PG at 1 h after birth. Methylation levels at these sites increased as neonatal PG levels at 1 h after birth decreased, suggesting that neonatal hypoglycaemia driven by the GDM status of mothers alters access to an alternative TSS of *ZNF696* in cord blood cells, which may be related to abnormal blood cell differentiation. Furthermore, epigenetic variations in offspring related to exposure to maternal hyperglycaemia, especially during early pregnancy, may reflect neonatal PG levels at 1 h after birth.

## Data Availability Statement

Data supporting the results of this article are available in the Gene Expression Omnibus repository (GSE122086, https://www.ncbi.nlm.nih.gov/geo/query/acc.cgi?acc=GSE122086 and GSE122288, https://www.ncbi.nlm.nih.gov/geo/query/acc.cgi?acc=GSE122288). Methylation data and analytical conditions are available upon request.

## Ethics Statement

The studies involving human participants were reviewed and approved by The Keio University School of Medicine Ethics Committee The Institutional Review Board of the National Research Institute for Child Health and Development. The patients/participants provided their written informed consent to participate in this study.

## Author Contributions

YK and KM collected the data; YK performed experiments; YK and TK performed statistical analyses, wrote the manuscript, contributed to the discussion, and reviewed/edited the manuscript; KM contributed to the discussion and wrote and reviewed/edited the manuscript; YS, SI, DO, MH, MTam, and KHas, MTan and KHat contributed to the discussion and reviewed/edited the manuscript. All authors contributed to the article and approved the submitted version.

## Funding

This study was supported by the Japan Agency for Medical Research and Development (18ek0109278h0002, 18ek0109290h0002, and 18mk0102093s0402), Japan Society for the Promotion of Science KAKENHI (17K19535 and 19K09761), and NCCHD of Japan research grant (2020B-21).

## Conflict of Interest

The authors declare that the research was conducted in the absence of any commercial or financial relationships that could be construed as a potential conflict of interest.

## Supplementary Material

Supplementary Figure 1 Quality evaluation of methylation data. **(A)** The results of cluster analysis of DNA methylation profiles in 132 neonates. **(B)** PCA was performed to confirm b values for the 132 samples to exclude outliers, resulting in exclusion of four samples.

Supplementary Figure 2 Collinearity assessment between blood cell types and PG at 1 h after birth. **(A)** The percentage of variances in each principal component were plotted following PCA of cell types and PG at 1 h after birth. The first principal component (PC1) explained 33.9% of the variations. **(B)** The contribution of each factor to the principal components is shown in the circled area. ‘Gran’ and ‘NK’ contributed the most to PC1, and PG at 1 h after birth contributed to PC3. Cell types contributed to different PCs. **(C)** Variable association plots. PG at 1 h after birth was positively associated with ‘Gran’ and ‘NK’ and negatively associated with ‘CD4T’.

Supplementary Figure 3 Correction of the genomic inflation factor (l). P-values were plotted using a Manhattan plot. QQ plots were plotted to show genomic inflation. Rlm analysis of DNA methylation in cord blood cells and continuous neonatal PG at 1 h after birth in 128 samples. Adding four covariates resulted in a l of 1.22. We considered PG at 1 h after birth to be significantly associated with DNA methylation sites after correcting the P-values with l (corrected to 1.1) (see **Figure 1B**).

Supplementary Figure 4 Association for each cell type with PG at 1 h after birth associated methylation sites. **(A)** Verification of the P-values for associations with DNA methylation at each of four CpG sites in the CpG island chr8:144371446–144372076 (hg19) and PG at 1 h after birth by adding each blood cell type individually as a covariate. Cell types not affecting the significance of the association between DNA methylation at cg11388673 and cg08799779. **(B)** QQ plots of analyses performed in **(A)**. Upper and lower rows indicate QQ plots before and after correction, respectively. **(C)** P-values following adjustment for the inflation factor and plotted with Manhattan plots. Data were derived from rlm analysis and adding individual cord blood cell types as covariates.

Supplementary Figure 5 The effect of each covariate on associations between DNA methylation and PG at 1 h after birth. Each Manhattan plot shows the P-values derived from rlm analysis after correcting with the inflation factor and the addition of each covariate confounder (neonatal sex, gestational age, and C-section) individually. The corrected inflation factor for each analysis is shown as a QQ plot. Neonatal sex was a major confounder of the association between DNA methylation at CpG sites in chr8:144371446–144372076 (hg19) and PG at 1 h after birth.

Supplementary Figure 6 The association between DNA methylation in 12 CpG sites proximal to the TSS of the ZNF696 isoform (ENST00000330143) and PG at 1 h after birth. Associations are shown according to neonatal sex.

Supplementary Figure 7 Reference data for TF ChIP-seq peaks from ENCODE3 data. Forty-six TFs bind the TSS of the ZNF696 isoform, whereas only ELF1 and RUNX3 were confirmed to bind the alternative TSS in the GM12878 cell line according to ENCODE3 data.

Supplementary Figure 8 Methylation values distribution at the 12 CpG sites in GDM and NGT were shown in box plot by neonatal sex. The 10 CpG sites whose methylation showed positive association with neonatal PG at 1 h after birth were indicated in upper panels above a dash line. The 2 CpG sites which showed negative association arrayed below a dashed line. Significant methylation value difference between GDM and NGT were analyzed by t-test (* p < 0.05).
