# Supplementary figures and images for: Epigenetic Changes in Neonates Born to Mothers With Gestational Diabetes Mellitus May Be Associated With Neonatal Hypoglycaemia
Source: Front Endocrinol (Lausanne). 2021 Jun 29;12:690648. doi: 10.3389/fendo.2021.690648 (PMC8276691; doi:10.3389/fendo.2021.690648)

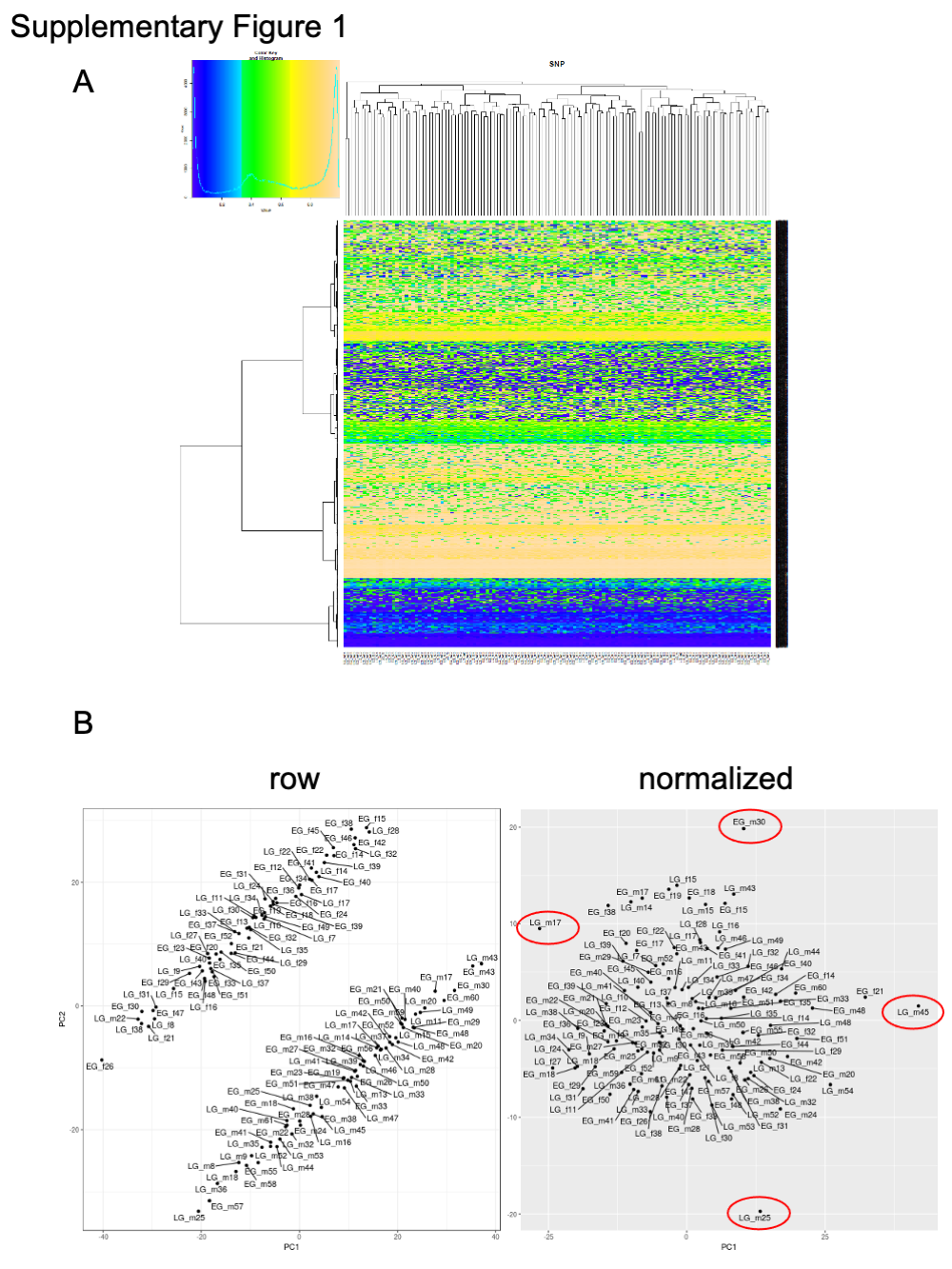

Supplement: Supplementary Figure 1 — Quality evaluation of methylation data. (A) The results of cluster analysis of DNA methylation profiles in 132 neonates. (B) PCA was performed to confirm b values for the 132 samples to exclude outliers, resulting in exclusion of four samples. [file Image_1.tiff]

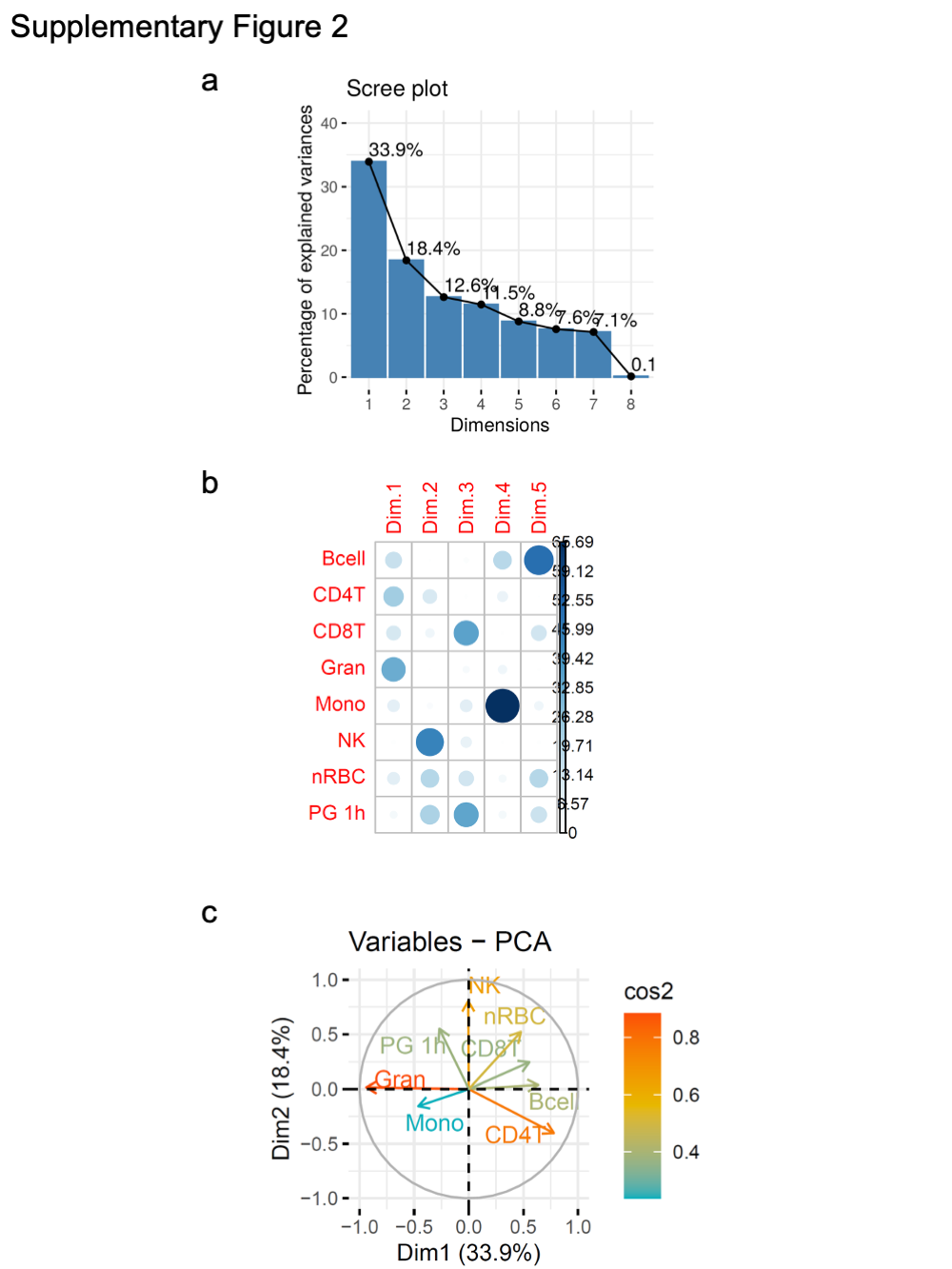

Supplement: Supplementary Figure 2 — Collinearity assessment between blood cell types and PG at 1 h after birth. (A) The percentage of variances in each principal component were plotted following PCA of cell types and PG at 1 h after birth. The first principal component (PC1) explained 33.9% of the variations. (B) The contribution of each factor to the principal components is shown in the circled area. ‘Gran’ and ‘NK’ contributed the most to PC1, and PG at 1 h after birth contributed to PC3. Cell types contributed to different PCs. (C) Variable association plots. PG at 1 h after birth was positively associated with ‘Gran’ and ‘NK’ and negatively associated with ‘CD4T’. [file Image_2.tiff]

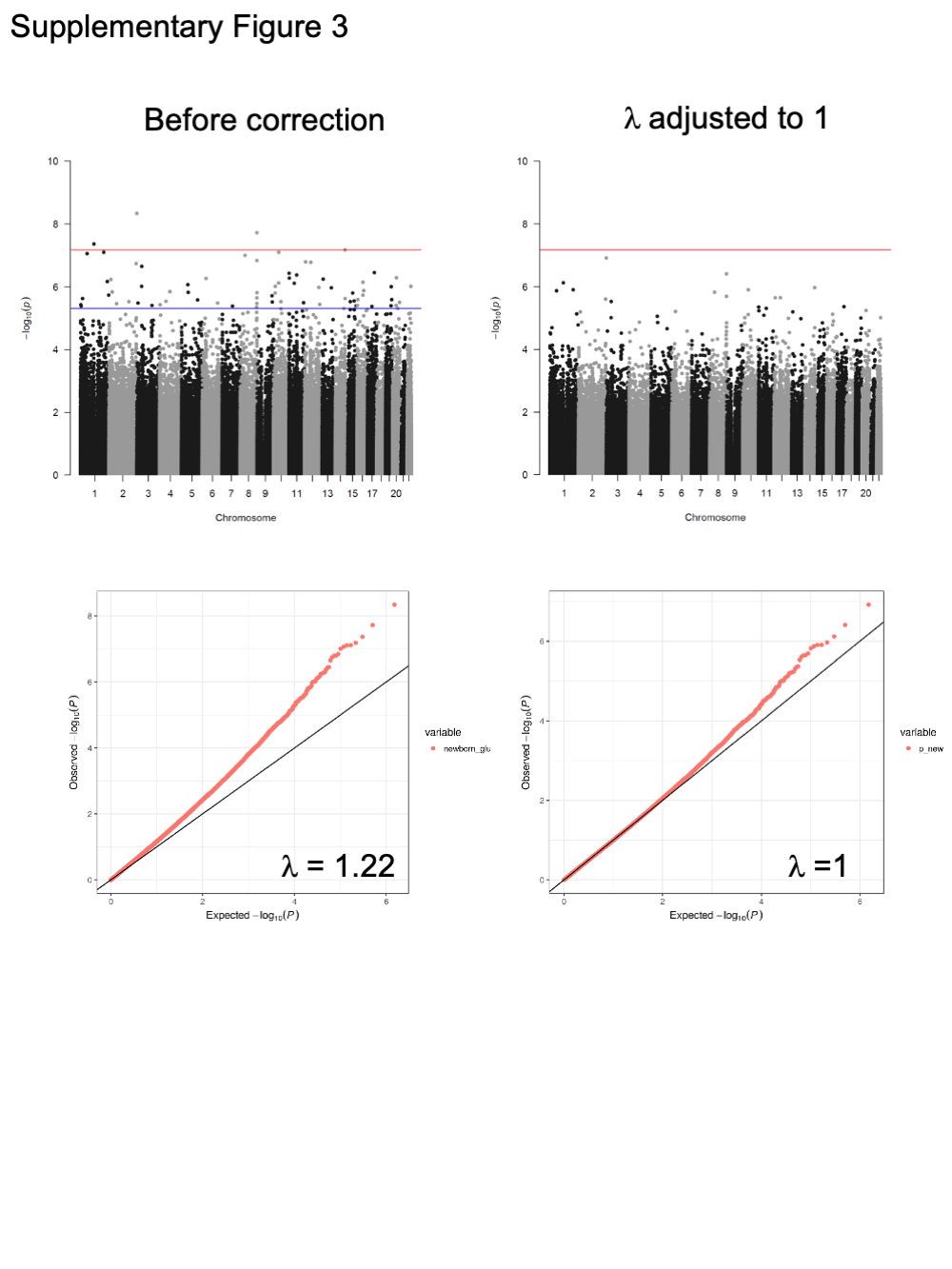

Supplement: Supplementary Figure 3 — Correction of the genomic inflation factor (l). P-values were plotted using a Manhattan plot. QQ plots were plotted to show genomic inflation. Rlm analysis of DNA methylation in cord blood cells and continuous neonatal PG at 1 h after birth in 128 samples. Adding four covariates resulted in a l of 1.22. We considered PG at 1 h after birth to be significantly associated with DNA methylation sites after correcting the P-values with l (corrected to 1.1) (see Figure 1B ). [file Image_3.tiff]

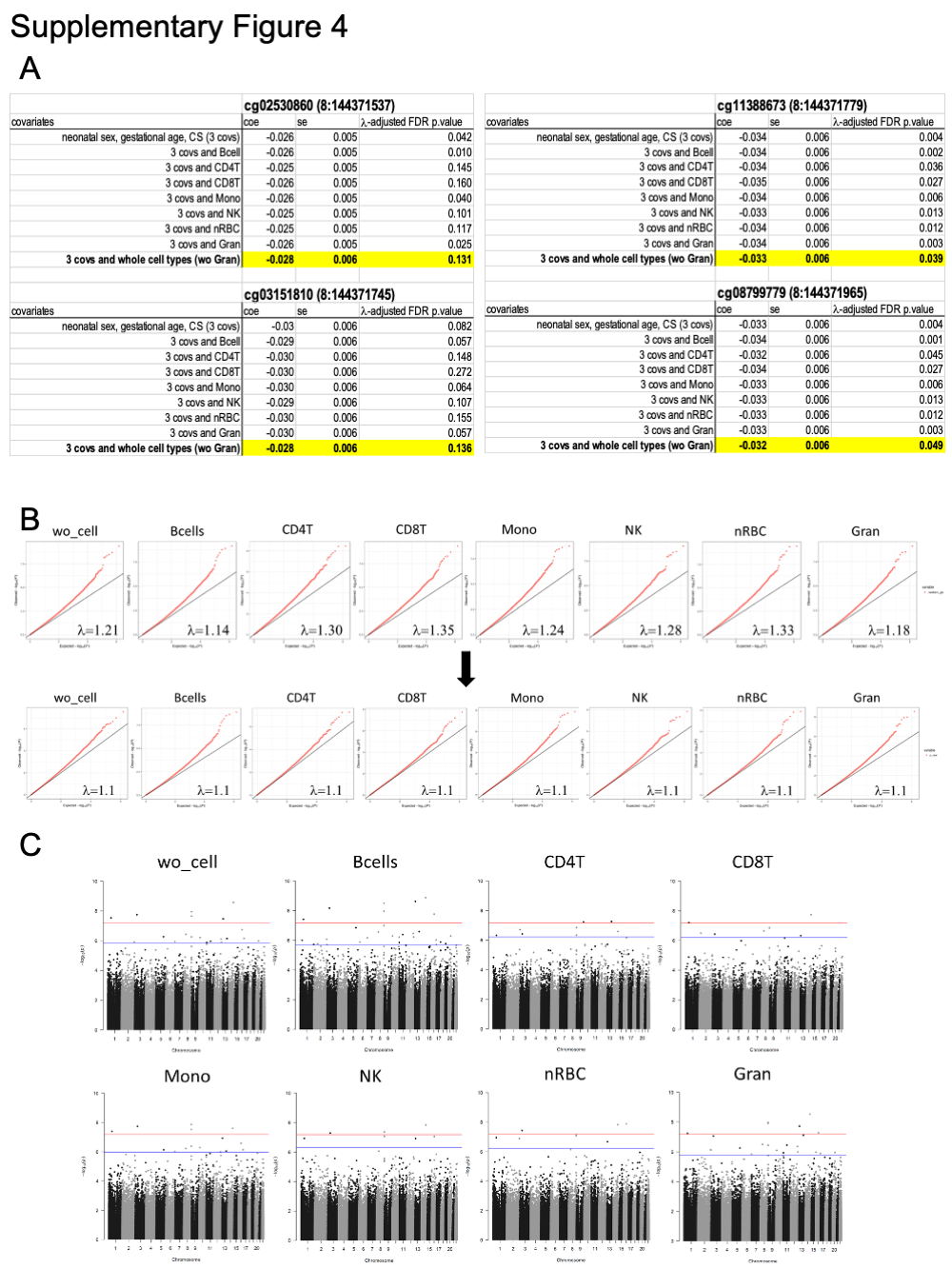

Supplement: Supplementary Figure 4 — Association for each cell type with PG at 1 h after birth associated methylation sites. (A) Verification of the P-values for associations with DNA methylation at each of four CpG sites in the CpG island chr8:144371446–144372076 (hg19) and PG at 1 h after birth by adding each blood cell type individually as a covariate. Cell types not affecting the significance of the association between DNA methylation at cg11388673 and cg08799779. (B) QQ plots of analyses performed in (A). Upper and lower rows indicate QQ plots before and after correction, respectively. (C) P-values following adjustment for the inflation factor and plotted with Manhattan plots. Data were derived from rlm analysis and adding individual cord blood cell types as covariates. [file Image_4.tiff]

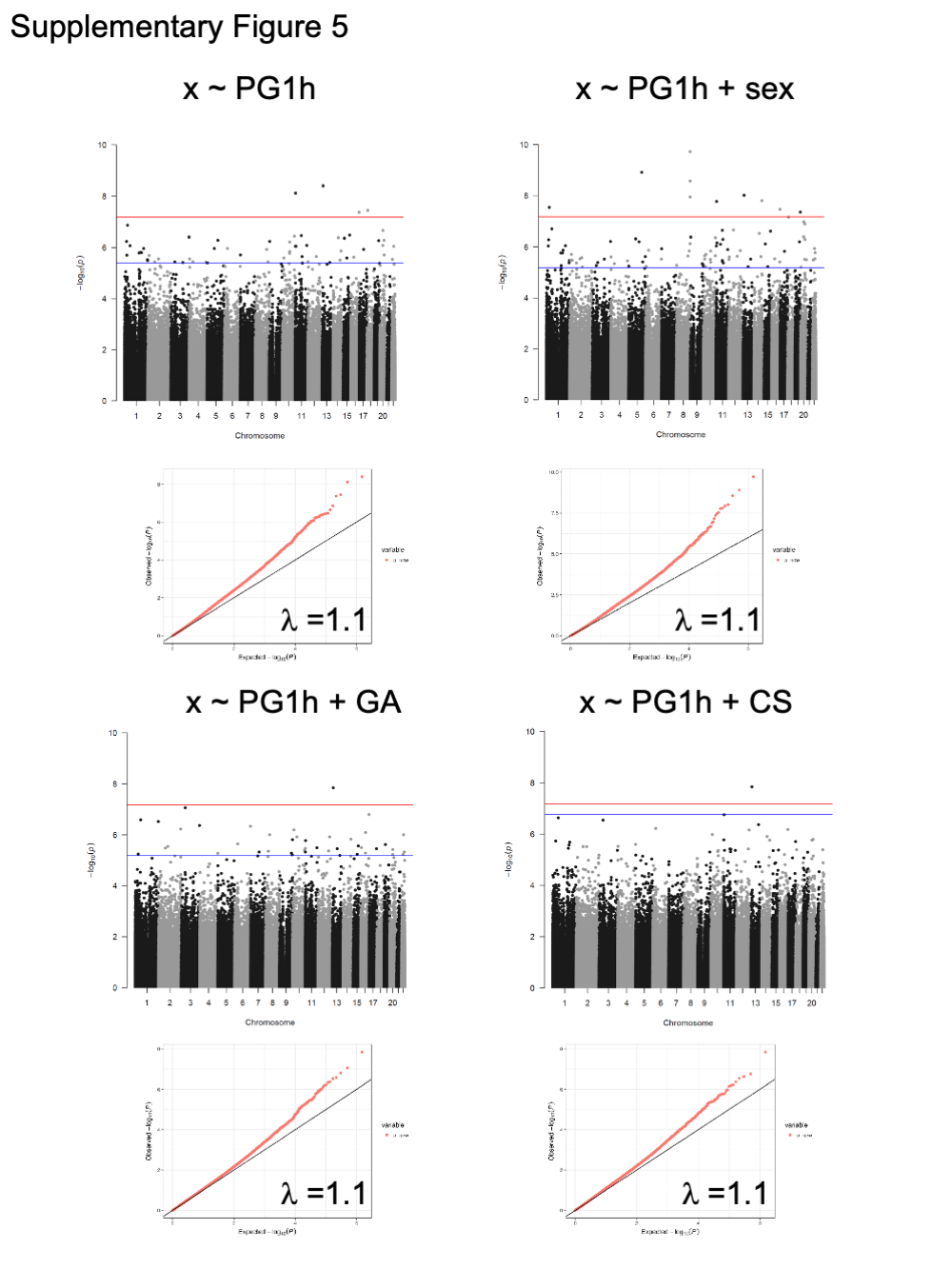

Supplement: Supplementary Figure 5 — The effect of each covariate on associations between DNA methylation and PG at 1 h after birth. Each Manhattan plot shows the P-values derived from rlm analysis after correcting with the inflation factor and the addition of each covariate confounder (neonatal sex, gestational age, and C-section) individually. The corrected inflation factor for each analysis is shown as a QQ plot. Neonatal sex was a major confounder of the association between DNA methylation at CpG sites in chr8:144371446–144372076 (hg19) and PG at 1 h after birth. [file Image_5.tiff]

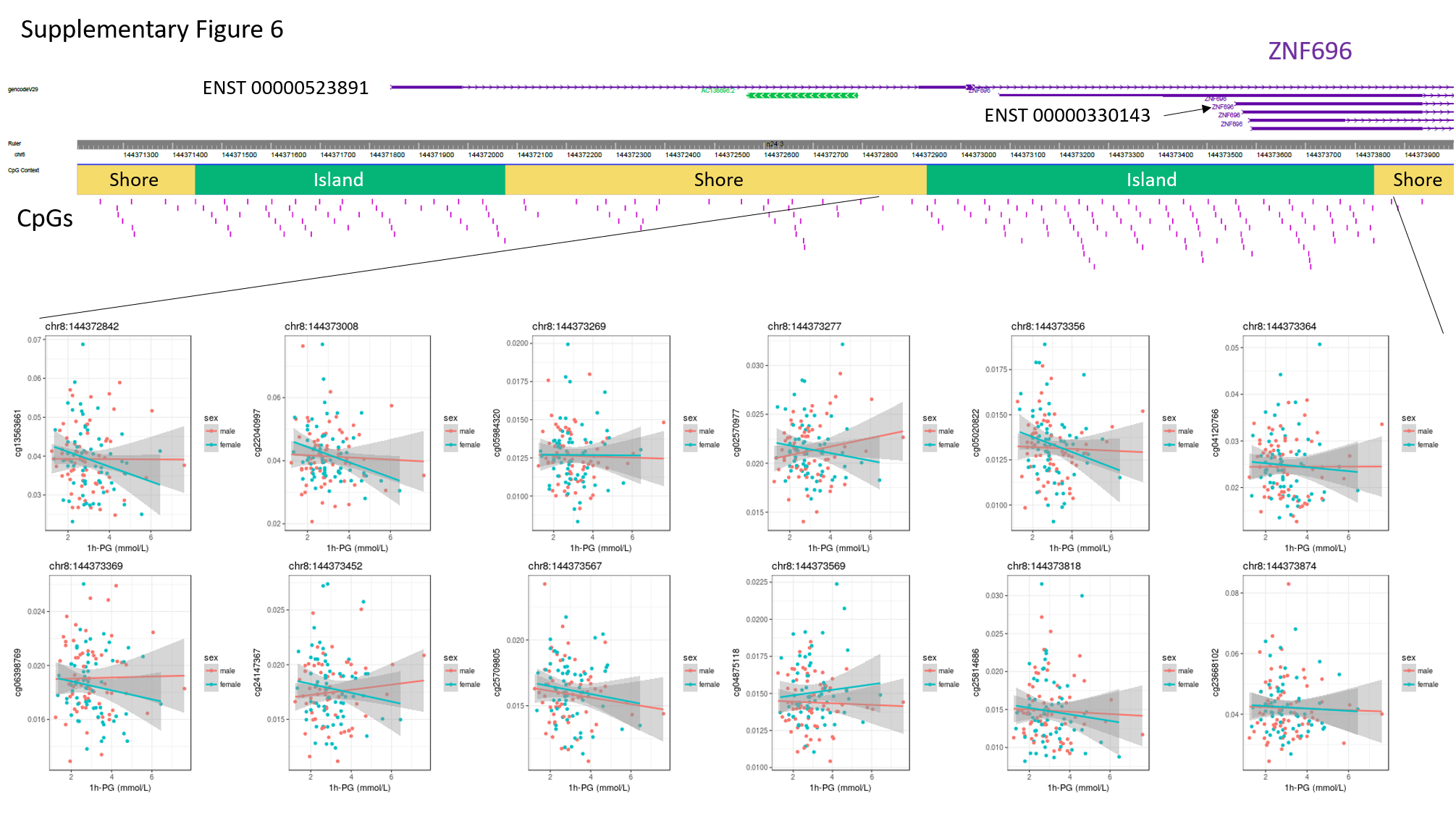

Supplement: Supplementary Figure 6 — The association between DNA methylation in 12 CpG sites proximal to the TSS of the ZNF696 isoform (ENST00000330143) and PG at 1 h after birth. Associations are shown according to neonatal sex. [file Image_6.tif]

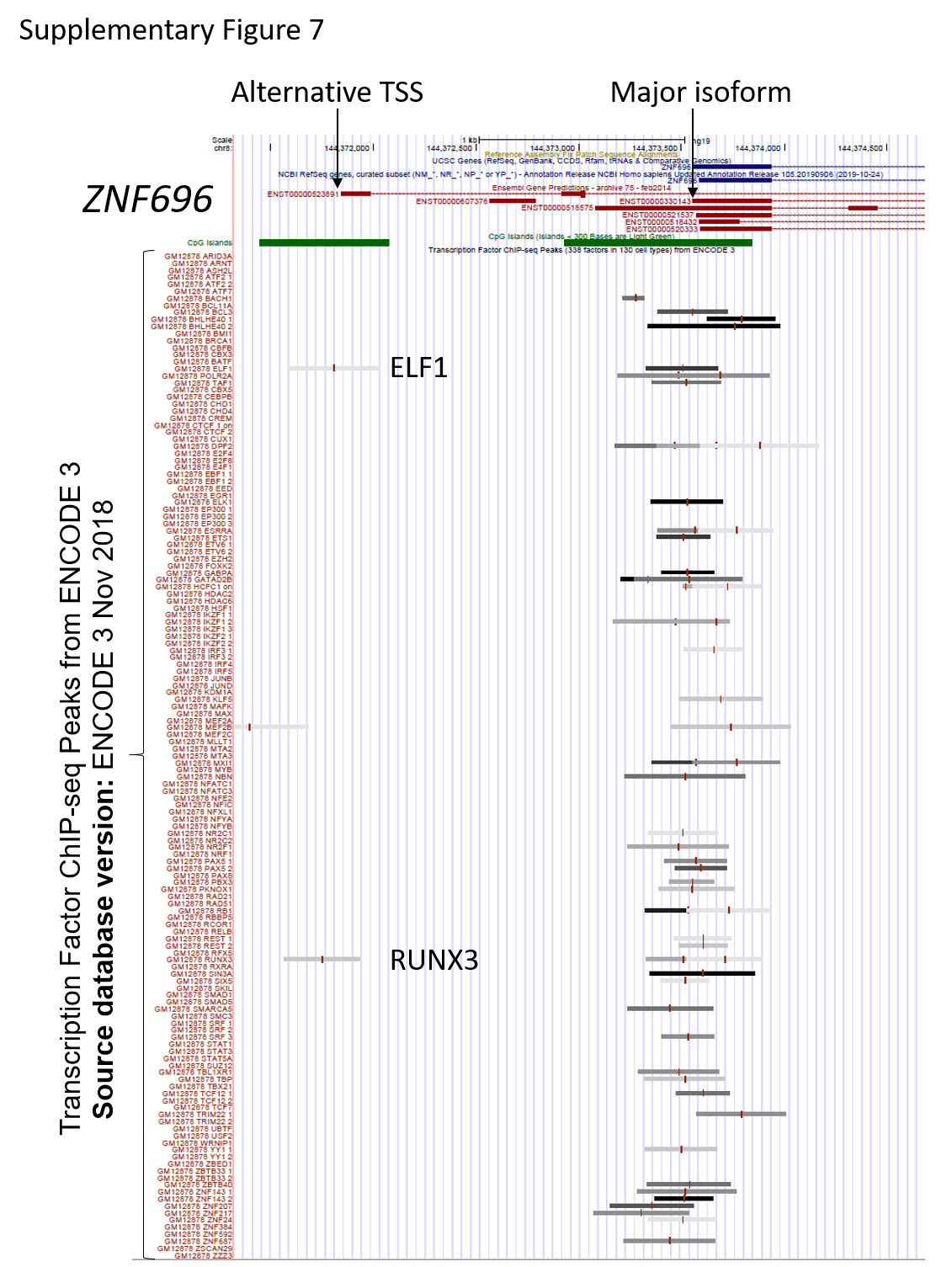

Supplement: Supplementary Figure 7 — Reference data for TF ChIP-seq peaks from ENCODE3 data. Forty-six TFs bind the TSS of the ZNF696 isoform, whereas only ELF1 and RUNX3 were confirmed to bind the alternative TSS in the GM12878 cell line according to ENCODE3 data. [file Image_7.tif]

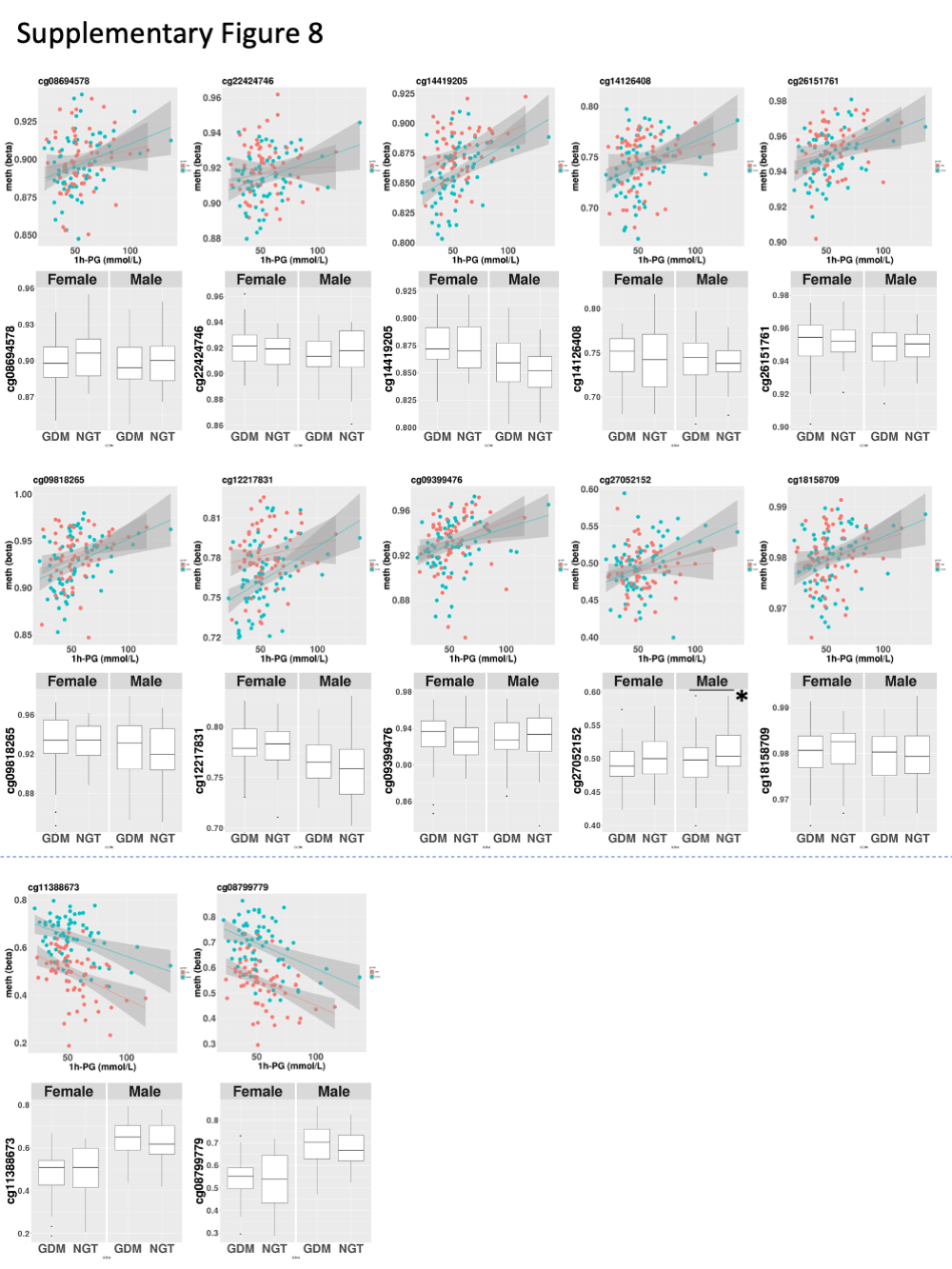

Supplement: Supplementary Figure 8 — Methylation values distribution at the 12 CpG sites in GDM and NGT were shown in box plot by neonatal sex. The 10 CpG sites whose methylation showed positive association with neonatal PG at 1 h after birth were indicated in upper panels above a dash line. The 2 CpG sites which showed negative association arrayed below a dashed line. Significant methylation value difference between GDM and NGT were analyzed by t-test (* p < 0.05). [file Image_8.tiff]
