# Supplementary material for: Epigenetic Changes in Neonates Born to Mothers With Gestational Diabetes Mellitus May Be Associated With Neonatal Hypoglycaemia
Source: Front Endocrinol (Lausanne). 2021 Jun 29;12:690648. doi: 10.3389/fendo.2021.690648 (PMC8276691; doi:10.3389/fendo.2021.690648)
Supplement: Supplementary file 9 [file Table_1.docx]

Supplementary Table 1. Comparison of maternal and neonatal characteristics between inclusion group (n=128) and exclusion group (n=4).

|  | Inclusion Group | | Exclusion Group | | p-value |
| --- | --- | --- | --- | --- | --- |
|  | (n=128) | | (n=4) | |  |
| Maternal age at delivery (years) | 37 | (26−47) | 38 | (33−42) | 0.60 |
| Maternal pregravid BMI (kg/m^2^) | 20.4 | (16.9–32.9) | 19.4 | (17.3−24.6) | 0.44 |
| Maternal insulin use during pregnancy | 33 | (26%) | 1 | (25%) | 1 |
| GA at delivery (weeks) | 39 | (37–41) | 38.5 | (38−40) | 1 |
| Caesarean section | 45 | (35%) | 1 | (25%) | 1 |
| Female neonates | 63 | (49%) | 0 | (0%) | 0.12 |
| Birth weight (g) | 3,022 | (2,352−3,834) | 3,005 | (2,550−3,202) | 0.60 |
| PG at 1 h after birth (mmol/L) | 2.8 | (1.2−7.6) | 2.95 | (1.6−4.1) | 0.90 |
| Hypoglycaemia (PG <2.6 mmol/L) | 45 | (35%) | 2 | (50%) | 0.62 |
| Umbilical artery pH | 7.31 | (7.13–7.44) | 7.3 | (7.28−7.40) | 0.70 |
| Apgar score |  |  |  |  |  |
| 1 min | 8 | (7–10) | 8.5 | (6−9) | 0.70 |
| 5 min | 9 | (7–10) | 9 | (9−10) | 0.66 |

BMI: body mass index, GW: gestational age, PG: plasma glucose level. Data are presented as median (range) or n (%). Continuous data were compared between groups using Mann-Whitney U test. Categorical variables were analyzed using Fisher’s exact test. In all tests, *P* < 0.05 was considered significant.
